# Mitochondrial DNA Evidence Indicates the Local Origin of Domestic Pigs in the Upstream Region of the Yangtze River

**DOI:** 10.1371/journal.pone.0051649

**Published:** 2012-12-13

**Authors:** Long Jin, Mingwang Zhang, Jideng Ma, Jie Zhang, Chaowei Zhou, Yingkai Liu, Tao Wang, An-an Jiang, Lei Chen, Jinyong Wang, Zhongrong Jiang, Li Zhu, Surong Shuai, Ruiqiang Li, Mingzhou Li, Xuewei Li

**Affiliations:** 1 Institute of Animal Genetics and Breeding, College of Animal Science and Technology, Sichuan Agricultural University, Ya’an, Sichuan, China; 2 Chongqing Academy of Animal Science, Chongqing, China; 3 Animal Husbandry Institute of Ganze Tibetan Autonomous Prefecture, Kangding, Sichuan, China; 4 Peking-Tsinghua Center for Life Sciences, Biodynamic Optical Imaging Center (BIOPIC) and School of Life Sciences, Peking University, Beijing, China; Onderstepoort Veterinary Institute, South Africa

## Abstract

Previous studies have indicated two main domestic pig dispersal routes in East Asia: one is from the Mekong region, through the upstream region of the Yangtze River (URYZ) to the middle and upstream regions of the Yellow River, the other is from the middle and downstream regions of the Yangtze River to the downstream region of the Yellow River, and then to northeast China. The URYZ was regarded as a passageway of the former dispersal route; however, this assumption remains to be further investigated. We therefore analyzed the hypervariable segements of mitochondrial DNA from 513 individual pigs mainly from Sichuan and the Tibet highlands and 1,394 publicly available sequences from domestic pigs and wild boars across Asia. From the phylogenetic tree, most of the samples fell into a mixed group that was difficult to distinguish by breed or geography. The total network analysis showed that the URYZ pigs possessed a dominant position in haplogroup A and domestic pigs shared the same core haplotype with the local wild boars, suggesting that pigs in group A were most likely derived from the URYZ pool. In addition, a region-wise network analysis determined that URYZ contains 42 haplotypes of which 22 are unique indicating the high diversity in this region. In conclusion, our findings confirmed that pigs from the URYZ were domesticated *in situ.*

## Introduction

Domestic pigs play an important role in the worldwide agricultural and economic sectors, and an understanding of the location, timing and process of pig domestication is essential to understanding the roots of modern civilization and the migratory trajectories that have shaped the modern geography of human language and culture [1]. The domestication of pigs is likely to have first occurred in the Near East approximately 9,000 years ago [2]. Genetic evidence based on mitochondrial DNA (mtDNA) sequences has revealed that Asian and European pigs were domesticated independently from local wild boar [3]. A worldwide phylogeography study of wild boar (*Sus scrofa*) demonstrated multiple centers of pig domestication across Eurasia [4]. Not only were Asian and European pigs proven to be domesticated *in situ* but domestication in local regions was shown to be common [1], [4], [5]. The first systematic study of complete mtDNA sequences between wild boar and domestic pigs across Asia revealed two main pig domestic centers in East Asia: the Mekong region and the middle and downstream region of the Yangtze River [5]. However, a recent study of Tibetan pigs indicated another pig domestication center in China - the Tibetan highland [6]. Thus, a region around China appears to have harbored multiple pig domestication events, similar to the multiple origin events documented as common phenomena in domestic animals such as cattle, goats, chickens, and horses [7]–[13].

The upstream region of the Yangtze River (URYZ), which contains the Sichuan, and Guizhou provinces and the Chongqing district, has an important role in archaeology, human history and civilization. The culture of Shu, a human civilization about 5,000 years old, mainly occurred in this region. Sanxingdui and Jinsha archaeological sites were also discovered in the URYZ. Domestic pig bones and pottery depicting pigs discovered in the Sichuan archaeological site are estimated to be approximately 5,000 years old and suggest a possible local domestication event in this region [14]. In addition, stone carvings from 2,000 years ago revealed prosperous pig cultivation in the URYZ [14]. In previous molecular studies, two main domestic pig dispersal routes in East Asia were indicated: one was from the Mekong region through the URYZ to the middle and upstream region of the Yellow River (MUYR); the other was from the middle and downstream regions of the Yangtze River (MDYZ) to the downstream region of the Yellow River (DRYR), and then to northeast China (NEC) [5]. This evidence indicated that the URYZ was likely a passageway for pig domestication; however, archeological and genetic evidence has shown conflicting results. It is reasonable to surmise that the number of individual samples used in previous studies of this region were limited, which may have obscured the conclusions. To resolve this, a more detailed sampling and analysis may be useful.

Here, we analyzed a large number of novel samples to investigate the domestication events in the URYZ. In this study, a commonly used hypervariable fragment in the mtDNA control region was used to analyze the differences in genetic diversity across a wide range of pig breeds in Asia. In total, 513 pigs were sampled from different districts mainly in Sichuan province, along with additional individuals from the Tibetan regions and from Jinhua, Zhejiang province. These regions contained five main Sichuan native breeds, one Zhejiang breed and six groups of Tibetan pigs in different geographic distributions. Another 1,394 sequences from regions across Asia that were deposited in the GenBank database were also analyzed. Therefore, this study uses the largest number of Sichuan pigs and pigs across Asia and provides the most comprehensive screening of mtDNA variations among domestic pigs and wild boars. The findings will help to increase our understanding of the matrilineal origin of domestic pigs in China.

## Materials and Methods

### Sampling and Sequencing

**Figure 1:**
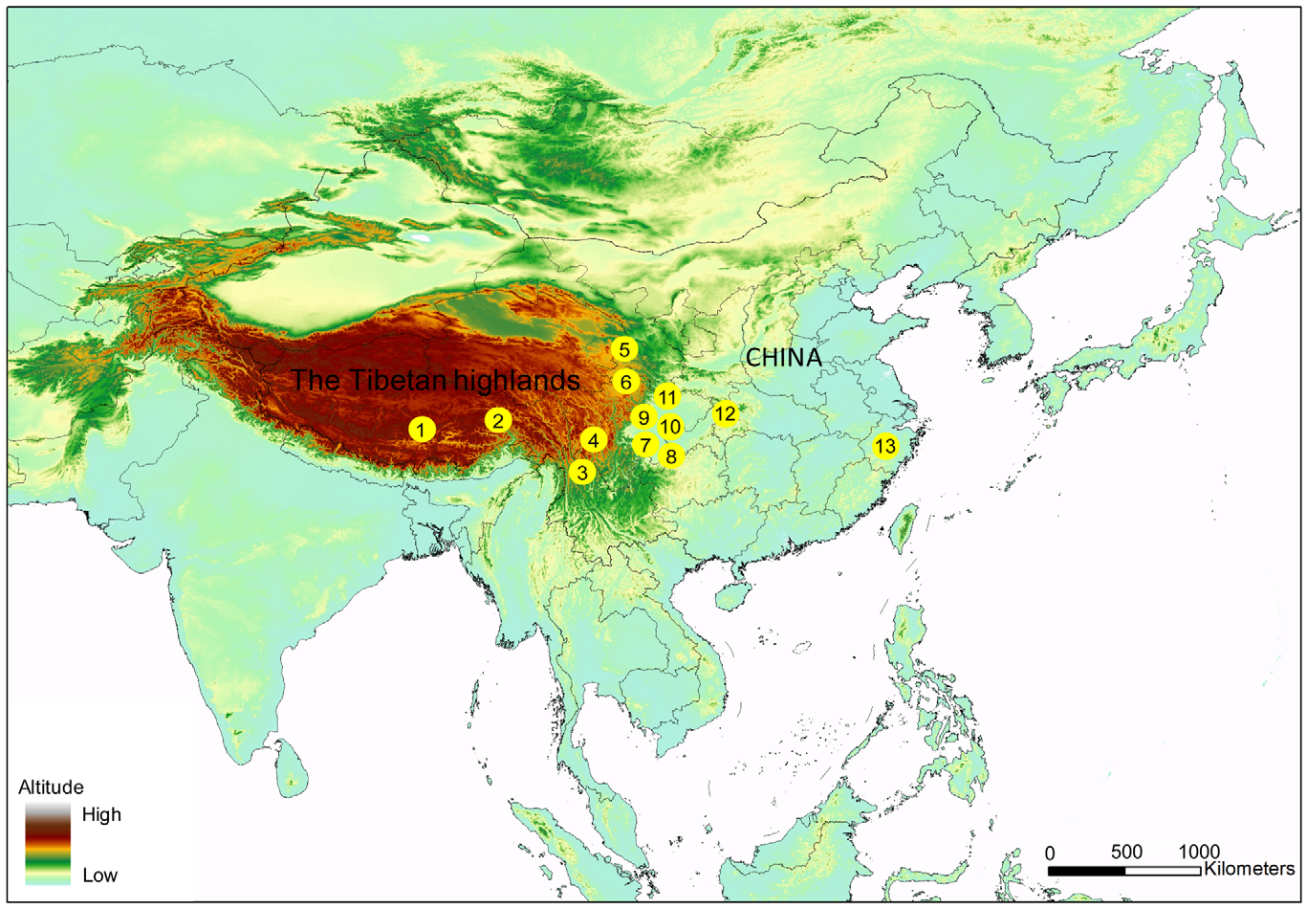
Sampling locations used in this study. Note: Codes 1–6 represent the Tibetan pig populations of Rikaze, Linzhi, Diqing, Ganzi, Hezuo and Aba, respectively. Codes 7–12 represent the Sichuan pig populations of Wu-jin, Pengzhou, Ya-nan, Neijiang, Chenghua and wild boars of Wushan. Code 13 represents the pig population of Jinhua.

All experimental procedures were approved by the Institutional Animal Care and Use Committee in College of Animal Science and Technology, Sichuan Agricultural University, Sichuan, China under permit No. DKY-B20100805. Only ear tissues were collected and preserved in ethanol 99% until extraction, and then animals were released immediately after treating wounds with antiseptic. Tissue samples of 513 individuals were collected from the Sichuan province (*n*  = 189), Tibetan highlands (*n*  = 274), and Zhejiang province (*n*  = 50) of China. All pigs used for sampling were identified with the distinct characteristics of pig breeds. Detailed animal samples were as follows: Sichuan native pigs (Wujin *n*  = 41, Pengzhou *n*  = 49, Ya’nan *n*  = 39, Neijiang *n*  = 49, Chenghua *n*  = 6), wild boar of Chongqing (*n*  = 5), Zhejiang jinhua pigs (*n*  = 50) and Tibetan pigs (Rikaze *n*  = 24, Linzhi *n*  = 96, Ganzi *n*  = 32, Aba *n*  = 36, Diqing *n*  = 38, Hezuo *n*  = 48) (Figure 1). DNA was isolated using the MicroElute Genomic DNA kit (OMEGA, USA). The D-loop region (approximately 680 bp) was amplified using the following primers previously published [4]: Forward 5′-CTCCGCCATCAGCACCCAAAG-3′; Reverse 5′-GCACCTTGTTTGGATTRTCG-3′. PCRs were set up using 25-µL volumes containing 1U taq polymerase, 500 µM dNTP, 20 Μm Tris-HCl (pH 8.3), 100 mM KCl, 3 mM MgCl_2_, and 10–100 ng DNA. PCRs were run under the following cycling conditions: 94°C for 5 min, followed by 40 cycles of 94°C for 30 s, 57°C for 30 s, 72°C for 1 min, and a final extension of 10 min at 72°C. PCR products were purified following agarose gel electrophoresis and then sequenced using the ABI 3730 DNA sequencer (Applied Biosystems, Foster City, CA, USA). All 513 sequences were submitted to GenBank, with reference numbers JX068012–JX068524 (Table S1).

### Analysis of Sequence Data

A total of 513 novel sequences were obtained through sequencing. Considering the quantitative limitation of our sequences, an additional 1,394 Asian pig sequences from previous studies were taken together to give a more comprehensive insight into the pig domestication history in Asia. All the downloaded sequences should be of approximately 680 bp in length. Thus, sequences that were significantly shorter than 680 bp were not used in our study, as all sequences used should contain as many variable sites as possible. Detailed information of all sequences is listed in Table S1.

Sequence data were edited using the DNASTAR software. All 1,907 sequences were aligned using Clustal X 1.81 [15]. In addition, MEGA 5.0 was used to confirm the alignments were correct [16]. To incorporate the shortest download sequence data, we reduced the length of alignment to 642 bp. The 642 bp fragments of mtDNA control regions were used to perform additional analyses. DNASP 5.0 software was used to analyze the haplotypes of all sequences and genetic diversity [17]. Gaps in the aligned sequences were excluded from this step and subsequent analyses. The African warthog (*Phacochoerus africanus*; GenBank accession number DQ409327) is distinct from the Eurasian wild boars, and was defined as an outgroup for phylogenetic analyses [5]. Before reconstruction of the phylogenetic tree, PAUP 4.0b10 and Modeltest 3.7 software were used to determine the optimum model and parameters [18]. Then the Bayesian consensus (MB) tree was constructed using the Monte Carlo Markov chain (MCMC) method by MrBayes 3.1 and the run was performed using 13 million generations [19]. All Maximum parsimony median-joining (MP) network plots were drawn using the software Network 4.6 [20].

All sequences used were partitioned into the 15 geographic regions referred to in previous studies: the URYZ, the MDYZ, the Tibetan highlands, Yunnan, South East Asia (SEA), Island South East Asia (ISEA), Pacific Islands, South Asia (SA), South China (SC), Taiwan, the MUYR, the DRYR, NEC, Japan and Korea [5], [6]. A detailed distribution of the 15 regions is shown in Figure S1 according to geographic fauna and possible pig domestication sites. From our novel sequences, Sichuan pigs and the Tibetan pigs were added to the URYZ and Tibetan regions, respectively. Additionally, Jinhua pigs of the Zhejiang province were regarded as a supplement to the MDYZ.

## Results

### Haplotype Analysis of Sequences

The 642 bp control region of mtDNA was used to calculate haplotypes for all 1,907 sequences (513 novel sequences and 1394 sequences derived from GenBank). No insertion/deletions (indels) were detected in our 513 novel sequences, whereas the downloaded 1394 sequences did harbor several indels compared with our data. And we excluded these indels for the alignment step and subsequent analyses. In total, 285 haplotypes were identified from the 1,907 samples, which contained 1,629 domestic pigs and 278 wild boars (Table S2). 189 haplotypes were found in domestic pigs and the wild boars contained 122 haplotypes. Twenty six haplotypes were shared by the domestic and wild pig populations. The sequence of haplotype one was used as a reference for alignment to discover variants (Table S2).

The genetic diversity of the domestic pigs across the 15 different regions was calculated (Table S3). The Korean region had the highest haplotype diversity (0.97±0.00196) and nucleotide diversity (0.0144±0.00157), while the NEC had the lowest haplotype diversity (0.48±0.00883) and nucleotide diversity (0.00207±0.00041). Meanwhile, SEA, Yunnan, URYZ and MUYR also had high haplotype diversities which of over 0.9. The haplotype diversity of Tibetan high land and MDYZ were 0.888±0.00013 and 0.875±0.00013, respectively. The small sample size of pigs from the Korean region may account for its apparent high genetic diversity. The number of domestic pigs from Japan and SA used in this study was small (six for both), therefore the genetic diversity of these two regions are not shown in Table S3.

### Phylogenetic Analysis

**Figure 2:**
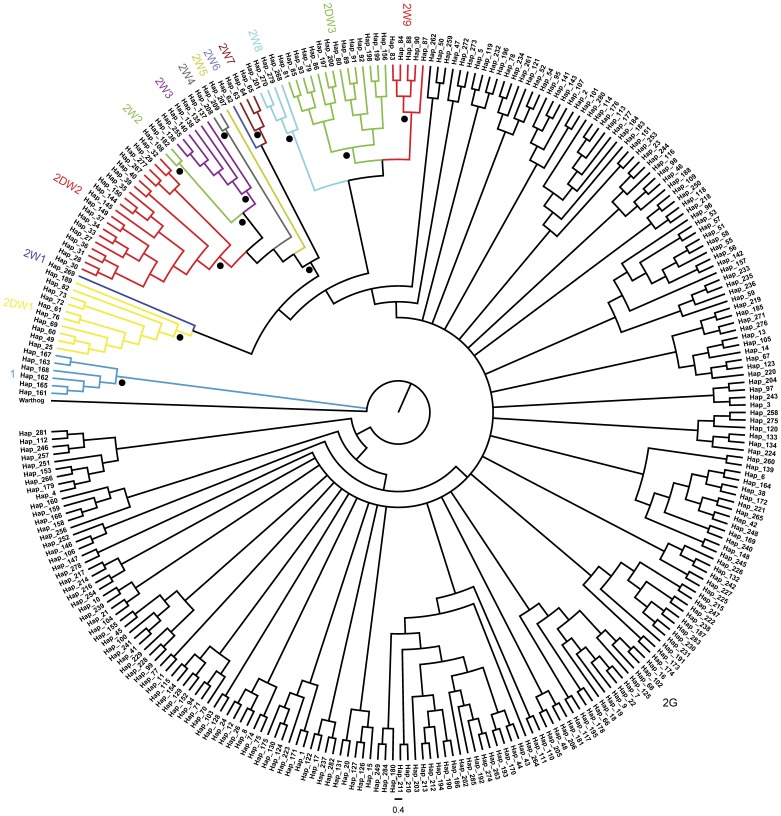
Bayesian consensus tree of 285 pig mtDNA control region haplotypes. Note: Branch color indicates the different clade designations. A dot indicates >65% posterior probability. A warthog was used for the outgroup. Detailed information of the haplotypes labeled at the end of each branch was listed in Table S2.

To study the relationship among pigs from different geographic regions, we used all 285 haplotypes to perform a phylogenetic analysis. The MB tree constructed using the MCMC method is shown in Figure 2. Only clade 1 could be easily identified. The rest of the haplotypes fell into a mixed group (clade 2), which had a low posterior probability (0.42). All the haplotypes in the mixed group were hard to distinguish by breed or geographical location. This result indicates that there were only small differences among individuals from the different regions across Asia. Although the mixed group is complicated, we could identify several subclades through the clear embranchments with high posterior probabilities (most were larger than 0.65). The remaining haplotypes in the mixed group could not be classified into subclades because of their low posterior probabilities. Therefore, all haplotypes were classified into 14 clades: 10 wild clades (clade 1 and clade 2 W1–clade 2 W9), three mixed clades which included both wild boars and domestic pigs (2DW1, 2DW2 and 2DW3) and clade 2G (a general group which contained pigs from across Asia).

To investigate the distribution of the clades, the number of haplotypes and individuals in each clade were calculated (Table 1). Among the 14 clades, clade 2G was the most frequent haplogroup, containing 235 haplotypes representing 1,759 individuals from across Asia. The 235 haplotypes included 166 domestic haplotypes and 69 wild haplotypes. Clade 1 contained six wild boars representing six haplotypes that were restricted to SA, suggesting that the wild boar population in this region is different from the wild boars in other regions. Among the wild groups, 2W1 only contained one wild boar from Yunnan. In addition, 2W8 was restricted to Yunnan and SEA and contained one wild boar from SEA and three wild boars from Yunnan; and 2W4 and 2W5 were restricted to SEA, and contained two individuals and one individual, respectively. Besides the unique wild haplotypes that were restricted to Yunnan and SEA, there were also three wild clades that were distributed in Japan and Korea. Subclades 2W6 and 2W7 contained only wild haplotypes that were restricted to Japan. Likewise, 2W9 contained seven individuals representing five unique wild haplotypes that existed only in Korea. Thus, in Japan and Korea, distinct wild boar lineages may exist. Six wild boars representing two haplotypes in subclade 2W2 were distributed in the MDYZ and SC. In addition, the major wild subclade 2W3 contained 19 individuals (six haplotypes) that were shared by the URYZ and MUYR. These subclades contained only wild boars suggesting that these wild populations may not have been used for domestication.

**Table 1:** Haplotype distribution between domestic pigs and wild boars.

Haplogroup	No. of haplotypes		No. of individuals		Region
	Domestic pig	Wild boar	Domestic pig	Wild boar	
1	–	6	–	6	SA
2DW1	9	1	14	1	SEA, DRYR, Korea, Japan
2W1	–	1	–	1	Yunnan
2DW2	13	8	43	10	Pacific Island, ISEA, SEA, Yunnan
2W2	–	2	–	6	MDYZ, SC
2W3	–	6	–	19	URYZ, MUYR
2W4	–	2	–	2	SEA
2W5	–	1	–	1	SEA
2W6	–	1	–	1	Japan
2W7	–	3	–	3	Japan
2W8	–	4	–	4	SEA, Yunnan
2DW3	1	13	1	29	SEA, Korea, SA
2W9	–	5	–	7	Korea
2G	166	69	1571	188	Throughout Asia

Note: (–) denotes that no sample was collected. The abbreviations for these regions are explained in the main text. Haplotypes used in statistics are referred to in Figure 2 and Table S2.

Three subclades that possessed haplotypes found in both wild and domestic samples were identified. The first clade, 2DW1, which contained mainly Korean and Japanese samples (six domestic samples from Korea, one wild sample and three domestic samples from Japan), but also included domestic individuals from SEA and the DRYR. The second clade, 2DW2, which contained 53 samples representing 21 haplotypes from samples that were mainly distributed in the Pacific Islands (32 domestic samples) and in ISEA (11 domestic and 7 wild samples), but also included several wild individuals from Yunnan. The third clade, 2DW3, contained only one domestic pig from SA and the rest of the wild individuals were all distributed in SEA and Korea. The subclades that contained samples from domestic pigs and wild boars demonstrate some small scale domestication events. In addition, not all wild boars share haplotypes with domestic pigs, suggesting that only certain types of wild boars were used for pig domestication. However, it was not possible to elucidate the likely domestication events for many regions across Asia from such an undefined phylogenetic tree pattern. Hence, network analysis was performed for further study.

### Network Analysis

**Figure 3:**
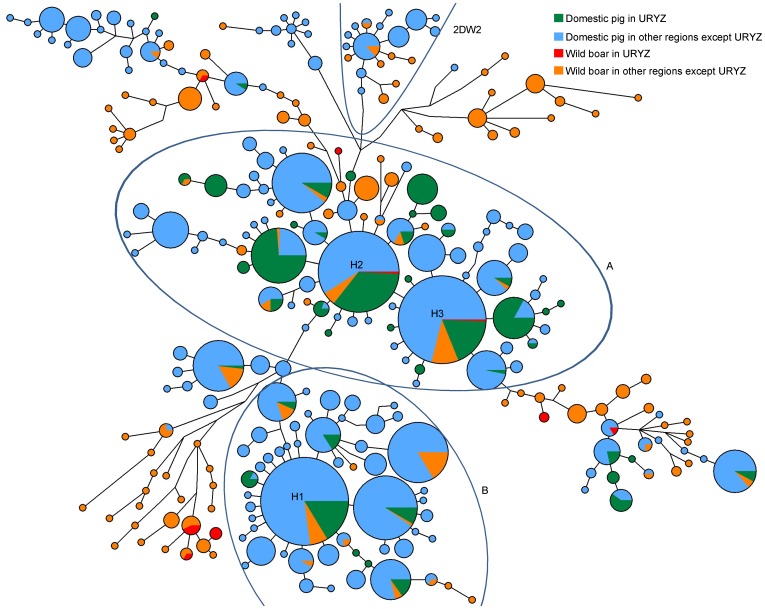
A Maximum parsimony median-joining network of domestic pigs and wild boars belonging to subclade 2. Colors within the nodes: green – domestic pig in URYZ; blue – domestic pig in other regions except URYZ; red - wild boar in URYZ; orange – wild boar in other regions except URYZ. Node sizes are proportional to haplotype frequencies. The link lines between nodes are proportional to the mutation steps. A and B labeled in the plot represent two haplogroups. 2DW2 represents the clade identified in Figure 2. H1, H2 and H3 labeled in the plot represent three core haplotypes: haplotype 1, haplotype 2 and haplotype 3, respectively.

In addition to the phylogenetic tree, we drew an entire network plot for clade 2 and 15 network plots of different regions (Figure 3 and Figure S2). The network plot for clade 2 still showed a complicated pattern. Nonetheless, on the periphery of the plot, several groups that were accordant with clades identified from the phylogenetic tree could be identified. Meanwhile, in the center region of the network plot, there were three core haplotypes, H1, H2 and H3 (each of which contained more than 100 individuals, Table S2), which covered most regions of Asia and were, without exception, shared by domestic pigs and wild boars. Each of the core haplotypes was surrounded by a star-like pattern, thus suggesting a recent population expansion had occurred (Figure 3).

To obtain more detailed information from the network, we defined two haplogroups (A and B) which consist of one or two frequently occurring core haplotypes surrounded by the less frequent haplotypes (Figure 3). Haplotypes that had many mutation steps were not included in the corresponding haplogroup. Haplogroup A contained the core haplotypes H2 (132 individuals, 15.2% of haplogroup A) and H3 (191 individuals, 20.9% of haplogroup A), and haplogroup B contains the core haplotype H1 (228 individuals, 36.5% of haplogroup B) (Figure 3, Table S4). In haplogroup A, of the total domestic pigs in this group, those from the URYZ had the highest frequency (227 individuals or 30.2%). A star-like pattern was found around H2 and H3; whereas, these two core haplotypes differed only by one mutation distance. Domestic pigs and wild boars from the URYZ shared two of the core haplotypes, H2 and H3. In addition, H2 contained one wild individual from the MDYZ and five wild individuals from Yunnan, and just one domestic pig from Yunnan and 47 domestic pigs from the MDYZ. Thirty-five domestic individuals from the URYZ had the highest frequency of this haplotype. A large number of samples from the Tibet highland were also found in this group, whereas no wild boar samples were collected from this region. Although there 47 domestic pigs from the MDYZ were in the H2, the number of domestic pigs from the MDYZ in group A was still lower than the number of domestic individuals of the URYZ. Taken together, this high frequency of URYZ individuals in haplogroup A strongly suggests that group A might have originated in the URYZ.

In group B, samples from the MDYZ had the highest frequency compared with all other regions (Table S4). The core haplotype H1 of haplogroup B contained 87 MDYZ individuals including 14 wild boars that shared a haplotype with domestic pigs, although there were also some individuals from other regions with this haplotype: DRYR (55 individuals), URYZ (37 individuals), Tibet (30 individuals), and SEA (8 individuals). Domestic pigs from the MDYZ had the highest frequency in haplogroup. Several derivatives that were one or two mutation distances from the core H1, which showed a star-like pattern, suggested that domestic pigs in group B were most likely derived from the MDYZ pool.

To investigate probable domestication events in the 15 separate regions, the numbers of haplotypes, including both domestic and wild haplotypes, from these regions were calculated (Table 2). From the network plots (Figure S2), five regions (URYZ, MDYZ, Tibetan highland, Yunnan and SEA) that have significantly higher haplotype numbers than other regions were clearly identified. A total of 323 samples in the URYZ, including 309 domestic and 14 wild samples were used to construct a network. Forty-three domestic haplotypes and nine wild haplotypes were found in this region, and two of the three core haplotypes of the URYZ were shared by domestic pigs and wild boars. Forty-nine haplotypes (40 domestic and 14 wild; five were shared by wild and domestic pigs) were identified in 318 individuals from the MDYZ. In all 327 samples from the Tibetan highland, 42 haplotypes were identified. Of the 51 haplotypes identified in samples from Yunnan, 28 domestic haplotypes were from 92 samples and 23 wild haplotypes were from 37 samples. In SEA, 48 haplotypes were found; 37 domestic haplotypes were from 151 samples and 11 wild haplotypes were from 13 samples. In addition, the network plot for each of these five regions harbor the three core haplotypes (H1, H2 and H3), each of which had several one or two mutation distance derivatives, except the plot from Yunnan that only harbored two of the three core haplotypes. Domestic pigs from the URYZ contained 18 unique haplotypes, and there were 20 one mutation derivatives around the core haplotype, which was the highest of the 15 regions. A similar high number of unique haplotypes was also found in domestic pigs from the MDYZ, SEA and the Tibetan highland. Although Yunnan contained 31 unique haplotypes, most were found in wild boars and only 12 were unique domestic haplotypes. It is interesting to observe that Yunnan, Korea and Japan harbored a large number of unique wild haplotypes. This high degree of endemism may result from a complex topography. The high diversity in wild boars of Japan may also be due to genetic divergence from mainland ancestors by disruption of gene flow. Collectively, the mtDNA data indicated that the URYZ, MDYZ, SEA and Tibetan highland are four diversity centers for domestic pigs.

**Table 2:** Regional distributions of haplotypes of domestic pigs and wild boars within subclade 2G.

Region	Domestic pig				Wild boar			
	Total	Nc (N)	N1 (N)	Nu (N)	Total	Nc (N)	N1 (N)	Nu (N)
Tibetan high land	42 (327)	3 (136)	14 (72)	18 (50)	–	–	–	–
Pacific Island	11 (49)	0	0	5 (30)	–	–	–	–
ISEA	18 (37)	1 (2)	1 (2)	11 (24)	6 (7)	0	0	6 (7)
SEA	37 (151)	3 (13)	10 (63)	18 (36)	11 (13)	0	2 (2)	9 (11)
Yunnan	28 (92)	2 (22)	5 (9)	12 (35)	23 (37)	2 (9)	3 (4)	19 (24)
URYZ	43 (309)	3 (118)	20 (146)	18 (56)	9 (14)	2 (2)	0	4 (7)
MDYZ	40 (283)	3 (129)	15 (39)	21 (40)	14 (35)	3 (19)	3 (3)	6 (9)
Taiwan	8 (18)	1 (3)	3 (4)	2 (2)	4 (5)	0	0	4 (5)
SC	20 (89)	2 (6)	8 (40)	8 (10)	15 (39)	1 (1)	2 (2)	7 (16)
MUYR	15 (45)	3 (9)	7 (14)	3 (16)	7 (15)	1 (1)	0	4 (8)
DRYR	25 (178)	3 (63)	8 (30)	11 (22)	-	0	0	–
Korean	11 (13)	1 (1)	2 (2)	10 (12)	19 (47)	0	1 (1)	18 (46)
Japan	6 (6)	0	0	3 (3)	16 (18)	0	0	16 (18)
NEC	3 (26)	1 (6)	0	0	6 (34)	1 (11)	0	4 (13)
SA	4 (6)	0	0	2 (2)	6 (8)	0	0	5 (7)

Note: (–) denotes that no sample was collected. Nc, number of core haplotypes; N1, number of one mutation distance derivatives; Nu, number of unique haplotypes. N in parentheses, number of individuals; DRYR: downstream region of the Yellow River; ISEA: Island South East Asia; NEC: northeast China; SA: South Asia; SEA: South East Asia; URYZ: upstream region of the Yangtze River; MDYZ: middle and downstream regions of the Yangtze River; SC: South China; MUYR: middle and upstream region of the Yellow River.

## Discussion

### Partial Domestication of Wild Boars in Asia

The wild boar has the widest geographical range of all ungulates and is one of the widest of all terrestrial mammals. It is native to Europe, Asia and North Africa with close to 25 classified subspecies [21], [22]. Previous studies indicated that pigs might have been domesticated independently from subspecies of the European and Asian wild boar populations, and this was also suggested by mtDNA analyses [23]. From the phylogenetic tree, we identified several wild subclades that were mainly distributed in SA, Korea, Japan, ISEA, SEA and China: the URYZ, MDYZ, Yunnan, MUYR and SC (Figure 2, Table 1). From the network analysis (Figure 3), most of the wild boar clades were found to be located at the periphery of the network plot, separated from the dominant clades in the center. Previous studies suggested that wild boar populations have a wide natural range [1]. This type of distribution would have provided preconditions for pig domestication [24], making it is reasonable to suppose that pig domestication in different regions could have begun with the local populations of wild boar [1]. Only some wild boars share haplotypes with domestic pigs (Figure 3), which suggests that wild pigs were only partially domesticated and the rest of the numerous populations did not contribute maternal genetic material to modern domestic pigs [1], [5].

### Pig Domestication in ISEA

The present study provides valuable insights into the origins of Asian pigs and the associated small scale domestication episodes through a comprehensive analysis of previously reported and new mtDNA data. From the phylogenetic tree, the matrilineal pool of East Asian domestic pigs and wild boars shows no clear differentiation of the regional breed/population pools (Figure 2). Meanwhile, some peripheral clades contain haplotypes shared by domestic and wild pigs, demonstrating that small scale pig domestication episodes occurred throughout Asia [4], [6]. Several studies have been performed on the origins of pigs from the Pacific Islands and ISEA [6], [25], [26]; however, no consensus had emerged regarding whether pigs from the Pacific Islands originated from SEA or ISEA. From our results, the clade 2DW2, which mainly contained individuals from ISEA and Pacific Island regions, exhibited a star-like pattern that is typical of exponential population growth (Figure 3). This clade harbored one core haplotype (Hap 27) that had a series of one, two, or greater than two mutation step derivatives (derived haplotypes) detected in domestic pigs and wild boars. Hap27 was shared by two wild boars in ISEA and 14 domestic pigs (12 from the Pacific Islands and two from ISEA). In addition, 2DW2 is a distinct monophyletic clade in the phylogenetic tree which contained samples and haplotypes mainly from ISEA and Pacific Islands, especially from Indonesian wild pigs and native domestic pigs (Figure 2, Table S2). Taken together, haplotypes within the 2DW2 subclade might have originated from the core haplotype (Hap 27) as the result of domestication events followed by subsequent expansion. The resulting pattern of mtDNA haplotypes show that domestic pigs in the Pacific Islands share a haplotype with wild boars in ISEA; thus, all our results strongly support the idea that pigs in the Pacific Islands originated from ISEA [6].

### Pig Domestication in China

Numerous genetic evidence has conclusively demonstrated that pigs were domesticated in East Asia, and China was shown to have two domestication centers: the MDYZ and the Tibet highland [5], [6]. Despite these findings, a lack of resolution and limitations of sampling has prevented refined conclusions to be made. Although the Tibetan pigs mainly distributed in the Tibetan highlands have adapted to the extreme environment and have been shown to have a local origin [6], our phylogenetic tree and network analysis results show that they are not distinguishable from others pig breeds in Asia. However, the Tibetan high land samples possess a great number of haplotypes or unique haplotypes and high genetic diversity (Table 2, Table S3), which is consistent with the local origin hypothesis of Tibetan pigs [6]. The data presented in this study demonstrate that all haplotypes in China are mixed in one big general group (2G), making it is difficult to distinguish them by different breeds or geography (Figure 2). This finding may be because of the lack of resolution of the D-loop regions probably caused by the lack of genetic differentiation between wild boar populations or gene flow among different regions.

URYZ and MDYZ individuals predominate in haplogroups A and B, respectively (Figure 3, Table S4). In these two regions, domestic pigs share a core haplotype with local wild boars. Previous studies identified two haplogroups, D1b and D1a2 [5], and the URYZ was not identified as a domestication center for pigs because the core haplotype in D1b and D1a2 was not shared by domestic pigs and wild boars. In contrast, we found that haplogroup A which contained the core haplotypes H2 and H3, was shared by most domestic pigs and wild boars from the URYZ, indicating that modern pigs in this region were domesticated from the local wild boar populations.

An additional 15 network plots for separated regions demonstrated that divergent diversities were found in the different regions. Direct comparisons of the geographic distribution between wild boars and domestic pigs can provide clues to clarify the domestication of East Asian pigs [5]. The number of derived or unique haplotypes was greatest in the URYZ, MDYZ, SEA, Yunnan and the Tibetan highland. In addition, these five regions had relatively higher haplotype diversity (Table S3), which is consistent with the great number of haplotypes. Outside these regions were the lower haplotype number and genetic diversity regions such as SC, NEC and DRYR (Figure S2 and Table S3). Shared haplotypes between domestic and wild pigs from the URYZ provided evidence which strongly suggested that URYZ pigs originated in the local region. Though Yunnan samples also had a high diversity, it showed an interspersed pattern without any dominant group in the total network (Table S2). Therefore, we regard Yunnan as a passageway for dispersal [6]. Previous studies have either indicated the URYZ as a passageway or have rarely discussed it as a major region because of the lower diversity that results from a limited sample size [5], [6]. Here, we reevaluated the URYZ and found it to be a potential diversity center. Thus, this study demonstrates that the domestication of pigs occurred mainly in the URYZ, MDYZ, SEA and Tibet [5], [6] and for the first time, the URYZ was found to be a potential diversity center.

Archaeological studies have discovered 5,000 year-old domestic pig dentary from Wushan County, Chongqing in the URYZ, suggesting that people in the URYZ had domesticated the pig in ancient China [14]. Pig domestication usually occurred due to various preconditions, such as the successful cultivation of certain cereals [24]. Previous archaeological studies have shown that rice cultivation existed in the upper Yangtze valley about 4000–5000 years ago [27]. Thus, it is likely that pigs were reared following the successful cultivation of rice, where any surplus could be used to feed pigs. Furthermore, at the archeological site in the URYZ, the discovery of pig bones suggests that domestication of pigs in the URYZ was common at that time, and may indicate that the local people cultivated numerous pig breeds around 2000 years ago. This conclusion is suggested by the differentiation of body size and morphology of many ceramic, stone, iron and silver pigs in the archaeological site [14]. In addition, epigeous stone carvings display prosperous domestic pig cultivation 2000 years ago in ancient China. The evidence based on genetics and archaeological studies indicates that domestic pigs of the URYZ have a local origin.

Numerous studies on archaeology, morphology, and domestication events of plants and animals revealed that the Yangtze River including the URYZ and MDYZ was a crucial area harboring many domestication events of rice, dogs and pigs [5], [27]–[31]. In this region, a process of rice domestication was found about 8200–13,500 years ago [28]. Dogs were shown to have a single origin to the south of the Yangtze River less than 16,300 years ago as determined by mtDNA data [29]. Pigs were also domesticated in the MDYZ [5] and URYZ, though the specific time for this domestication event has not been established. Thus, the river valley, Yangtze River and Yellow River in China, have played important roles at the beginning and in the development of human civilization, from hunting to sedentary pursuits and the subsequent domestication of plants and animals [32].

In summary, our results indicate that pigs from the URYZ were domesticated *in situ* and support the idea of multiple origins of domestic pigs in China. However, the conclusion is inferred from maternal genetic material of mtDNA and there are still some difficulties in distinguishing the different breeds or populations. Therefore, it is necessary to test this hypothesis through a detailed study of pigs in Asia using zoo archeological records, ancient DNA, and more wild pig samples from more locations, and through the use of more accurate molecular methods.

## Supporting Information

Figure S1 **Geographical and group distribution of all samples used in this study.**
(TIF)

Figure S2 **Maximum parsimony median-joining networks of 15 regions.** (A) URYZ, (B) MDYZ, (C) the Tibet highland, (D) Yunnan, (E) DRYR, (F) MUYR, (G) SEA, (H) ISEA, (I) Japan, (J) Korea, (K) NEC, (L) Pacific Islands, (M) SA, (N) SC, (O) Taiwan. Colors within the nodes: blue - domestic; orange - wild. Node sizes are proportional to haplotype frequencies. The link lines between nodes are proportional to the mutation steps. Core haplotypes are indicated with bold lining.
(TIF)

Table S1 Information on sampling of Asian domestic pigs and wild boars.
(XLS)

Table S2 Haplotype and geographical distributions across 15 regions.
(XLS)

Table S3 Genetic diversity of domestic pigs across 15 different regions.
(XLS)

Table S4 The geographic distribution of Asian domestic pigs and wild boars within haplogroup A, B and core haplotypes. Note: (-) denotes that no sample was collected. A and B: haplogroup; H1, H2 and H3: core haplotype. The values in parentheses refer to the proportion of individuals that are grouped into this haplogroup or core haplotype.
(XLS)
