# Supplementary figures and images for: Mitochondrial DNA Evidence Indicates the Local Origin of Domestic Pigs in the Upstream Region of the Yangtze River
Source: PLoS One. 2012 Dec 13;7(12):e51649. doi: 10.1371/journal.pone.0051649 (PMC3521662; doi:10.1371/journal.pone.0051649)

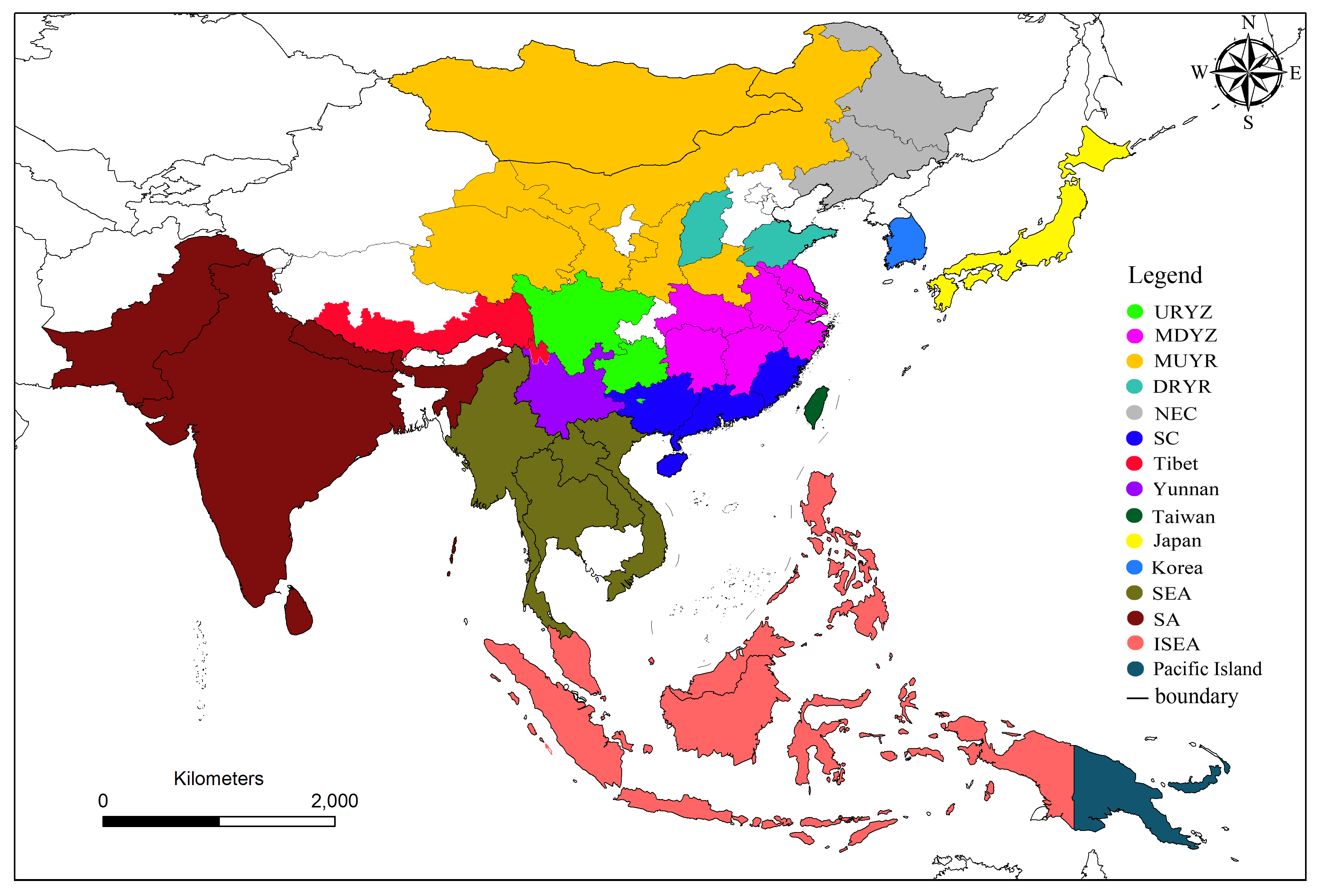

Supplement: Figure S1 — Geographical and group distribution of all samples used in this study. (TIF) [file pone.0051649.s001.tif]

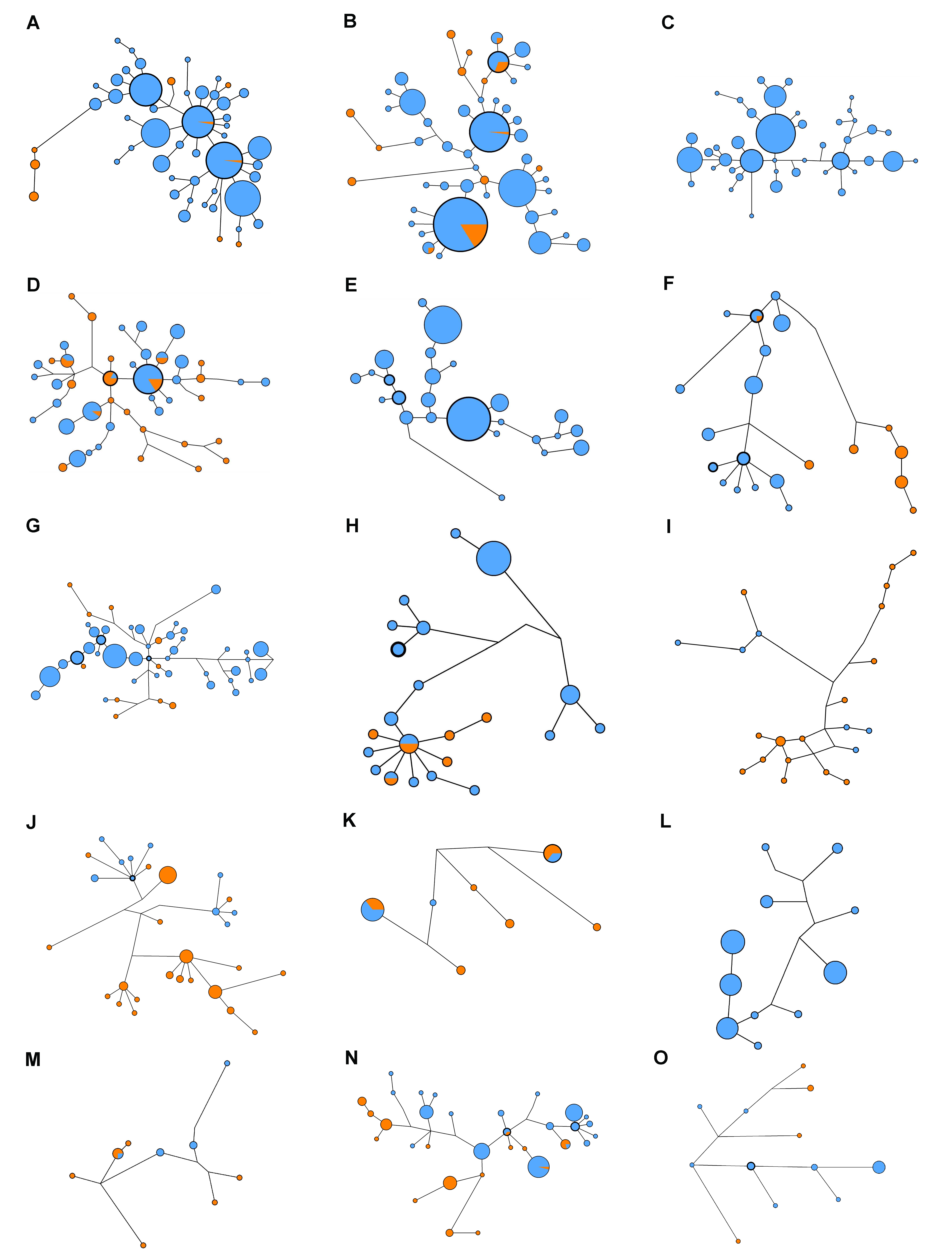

Supplement: Figure S2 — Maximum parsimony median-joining networks of 15 regions. (A) URYZ, (B) MDYZ, (C) the Tibet highland, (D) Yunnan, (E) DRYR, (F) MUYR, (G) SEA, (H) ISEA, (I) Japan, (J) Korea, (K) NEC, (L) Pacific Islands, (M) SA, (N) SC, (O) Taiwan. Colors within the nodes: blue - domestic; orange - wild. Node sizes are proportional to haplotype frequencies. The link lines between nodes are proportional to the mutation steps. Core haplotypes are indicated with bold lining. (TIF) [file pone.0051649.s002.tif]
